# Digital twin models of replicative ground stones: insight into simulating usage of Upper Paleolithic tools

**DOI:** 10.1038/s41598-023-45425-4

**Published:** 2023-10-25

**Authors:** Maria Rosaria Marulli, Giusi Sorrentino, Fabio Menna, Marco Paggi

**Affiliations:** 1 IMT School for Advanced Studies Lucca, Piazza San Francesco 19, 55100 Lucca, Italy; 2 Department of Physics, University of Turin, Via P. Giuria 1, 10125 Turin, Italy; 3 3D Optical Metrology Unit, Bruno Kessler Foundation (FBK), Via Sommarive 18, Povo, 38123 Trento, Italy

**Keywords:** Archaeology, Computational science, Engineering

## Abstract

This work presents the first attempt to create a physics-based digital twin model for predictive analysis of damage evolution during the use of ground stone tools (GSTs) in transformative tasks, encompassing the processing of raw resources for nutritional and non-alimentary purposes. The proposed methodology introduces a digital twin of the GSTs developed from 3D models generated using a photogrammetric technique based on Structure-from-Motion and Multi-View Stereo reconstruction. These models serve as the foundation for the development of the finite element (FE)-based digital twin model of the GSTs that exploits a contact formulation and the phase-field approach to simulate tool damage during pounding and grinding tasks. Defining the initial relative positions of the stones, their mechanical behaviour, and controlling the movement of the active stone in a way as close as possible to the real one, the digital twin model has been devised to evaluate how the surface damage is affected by perturbations in the loading conditions. The simulated damage is compared with the surface traces observed from experiments. The developed digital twin model aims at demonstrating its potentials for the GSTs investigations, as a supporting tool for experiments and for simulated tests on the archaeological records.

## Introduction

This work exploits the potential of digital twin model and predictive analysis for ground stone tools (GSTs) studies. GSTs represent a category of artefacts that encompass a diverse range of domestic toolkits manufactured and/or used across various daily activities. These tasks involve the processing of different raw materials for various purposes. Examples of such activities include the production of mineral pigments, bone fragmentation, nut cracking, wood pounding for fibre extraction, plant grinding for flour production, leather preparation, ceramic vase polishing, and numerous other tasks that necessitate actions such as percussion, pounding, cracking, grinding, abrasion, polishing, chopping, pecking, and softening (e.g.,^1–3^). In this study the focus is on unmodified stones, including cobbles, river pebbles, and slabs, used by Anatomically Modern Humans during the Upper Palaeolithic to process plant organs, for various nutritional and non-alimentary purposes. This involved transforming them into flour, softening them for easier chewing and digestion, or separating fibres to create treats. Typically, Upper Palaeolithic GSTs used in plant processing were used in pairs, with a lower steady passive stone where the resource to be processed was placed, and an active, movable tool held in one hand for grinding, pounding, and pulverising tasks.

Experimental reproduction, through repeated trials, can be exploited to gain insights and enhance the understanding of the kinetics of tools, specifically the underlying motion and forces that contribute to the formation of use-wear traces. By replicating and closely observing the motion and forces involved in the use of these tools, researchers can uncover valuable information about the wear patterns and the mechanisms and processes involved in their formation. Additionally, the tribological mechanisms that occur when the two lithic surfaces and the medium come into contact can be analysed and used as a reference for a deeper understanding of the functional aspects of the GSTs and the activities they were employed for (e.g.,^2,4^, pp: 27–41,^5,6^).

When designing an experimental ground stone collection, various approaches can be employed, which have been classified and categorised by scholars based on different parameters^3–8^. What is of interest for this research is the degree of control over variables that these different approaches allow^9^. Therefore, we took advantage of the finality of actualistic experiments (or what Marreiros and colleagues defined as first-generation experiments in 2020^8^) to determine the tools' applicability, efficiency, and durability in specific tasks by attempting to replicate ancient technology and gestures. However, this was carried out in a laboratory setting to control and verify the impact of variables in the experiment^9^ and to ensure a high level of internal validity^10^. As emphasised in^11^, we cannot fully recreate or reconstruct prehistory because nobody truly knows how it was. Nevertheless, to address the concern raised by Lycett and Eren in 2013^10^ regarding the low external validity of experimental replicas, our collection was carefully designed with control over morphometric and petrographic characteristics to ensure compatibility with archaeological contexts^9^.

In the GSTs study, a higher internal validity can be achieved in mechanical setups within laboratory-controlled environments (e.g.,^12–14^). These setups allow for reproducibility and offer control over factors that are challenging to monitor in a manual experiment, such as the number of percussions, their direction, and the applied pressure^15–19^. However, it is fundamental to acknowledge that the design of the machinery itself can introduce variables that may not exist in actions performed by humans and may also exclude the introduction of variabilities that occur during manual use, moving away from the characteristics of real archaeological records. Therefore, to enhance the level of internal validity without compromising the connection to the relevant archaeological context, we also explore different approaches involving mathematical and mechanical models that can be controlled and manipulated with high precision. With this scope, the present work proposes a new path for the GSTs analysis that has not been attempted so far: developing a finite element (FE)-based digital twin model to investigate their past usage.

The digital twin (DT) model can be, in general, defined as a physics-based dynamic computer representation of an actual item, which includes the historical information, geometrical mechanical and structural characteristics of a physical object, and simulates how the virtual model reacts to external stimuli^20^. It has been recently proposed in cultural heritage for preserving and protecting works of art, museum assets, historical buildings, and archaeological sites (e.g.^21–24^).

In the present work, we modelled the mechanical behaviour and the damage of the GSTs using the FE method^25,26^, which is traditionally applied to engineering problems including structural analysis, heat transfer, fluid flow, mass transport, etc. In fact, the deformation of solids exposed to mechanical loads, as well as their interactions, can be mathematically described using *partial differential equations* (PDEs), accounting for the external constraints to which the solids are subjected and their mechanical properties. Analytical solutions of the PDEs become very difficult to achieve for complicated shapes. However, a numerical approximation of the solution can be achieved by discretizing the structural system in small units called "finite elements" to obtain the solids mesh. In this way, the FE method allows the solution of the PDE, computation of displacement and force distributions within the solids. More details can be found in the Supplementary document.

Examples of FE models of archaeological records can be found in the research work^27^ dealing with Clovis projectile points and aiming at understanding the role of fluting. The projectile points were modelled using a model geometry not acquired from the real tool. The same approach is considered in^28^, where 3D models of arrowheads belonging to the Turk Khaganate, one of the foremost cultures in Eurasia, were simulated striking an armour plate belonging to this period. Other applications can be found in^29,30^, where Hellenist ceramic amphoras have been simulated to interpret damage and failure patterns found in the archaeological assets. The FE model has been used to simulate their stacking in cargo ships and understand how the mechanical loads and the contact interactions could cause fracture propagation. Damage was estimated based on a linear elastic stress analysis, by comparing the obtained stress distributions within the amphoras with the experimental ceramics' strength.

In this work, a more advanced approach to evaluate the damage is proposed, the so-called *phase-field variational approach (e.g., *^31–33^*)*, which introduces a further unknown variable in the PDEs describing the solids mechanics, i.e., the phase-field variable taking values between 0, corresponding to the intact material, and 1 where the material is broken. This method has been applied in the present investigation to reproduce the evolution of damage, and eventually material removal, of the tools’ surfaces during the contact process. The authors coded the phase-field governing equations within the commercial FE software Abaqus (ver. 6.14) with a user-defined routine, as detailed in the Supplementary. To the authors' best knowledge, this promising technology has never been applied to archaeological items, and even in the engineering field the use of the phase-field method within contact-induced fracture problems has never been attempted for complex 3D geometries in contact (see^34^ and references therein).

This study aims to develop a GSTs digital twin model that reproduces accurately the stone tools’ geometry and exploits a high-fidelity mechanical model that simulates the tribological behaviour and the damage pattern left on the surfaces during the tools' usage. The proposed DT model can be used to investigate the original usage of these artefacts in substitution or conjunction with the existing research strategies described above.

Starting from acquiring the stones' shape through optical methodologies, the DT model has been exploited to simulate the grinding tasks, defining the initial relative positions of the stones, their mechanical behaviour, and controlling the movement of the active stone during the act to reproduce the real one. The DT model offers the same advantages as the laboratory's experimental tests but can also investigate the role of the variability typical of human actions. Additionally, it can reduce contamination by comprehending how the experimental design affects the outcomes. Moreover, while the traditional approaches can be applied only to experimental replicas, the digital twin model can be directly constructed on the original archaeological artefacts, starting from their digital models, and following the same procedure described in this paper.

**Figure 1:**
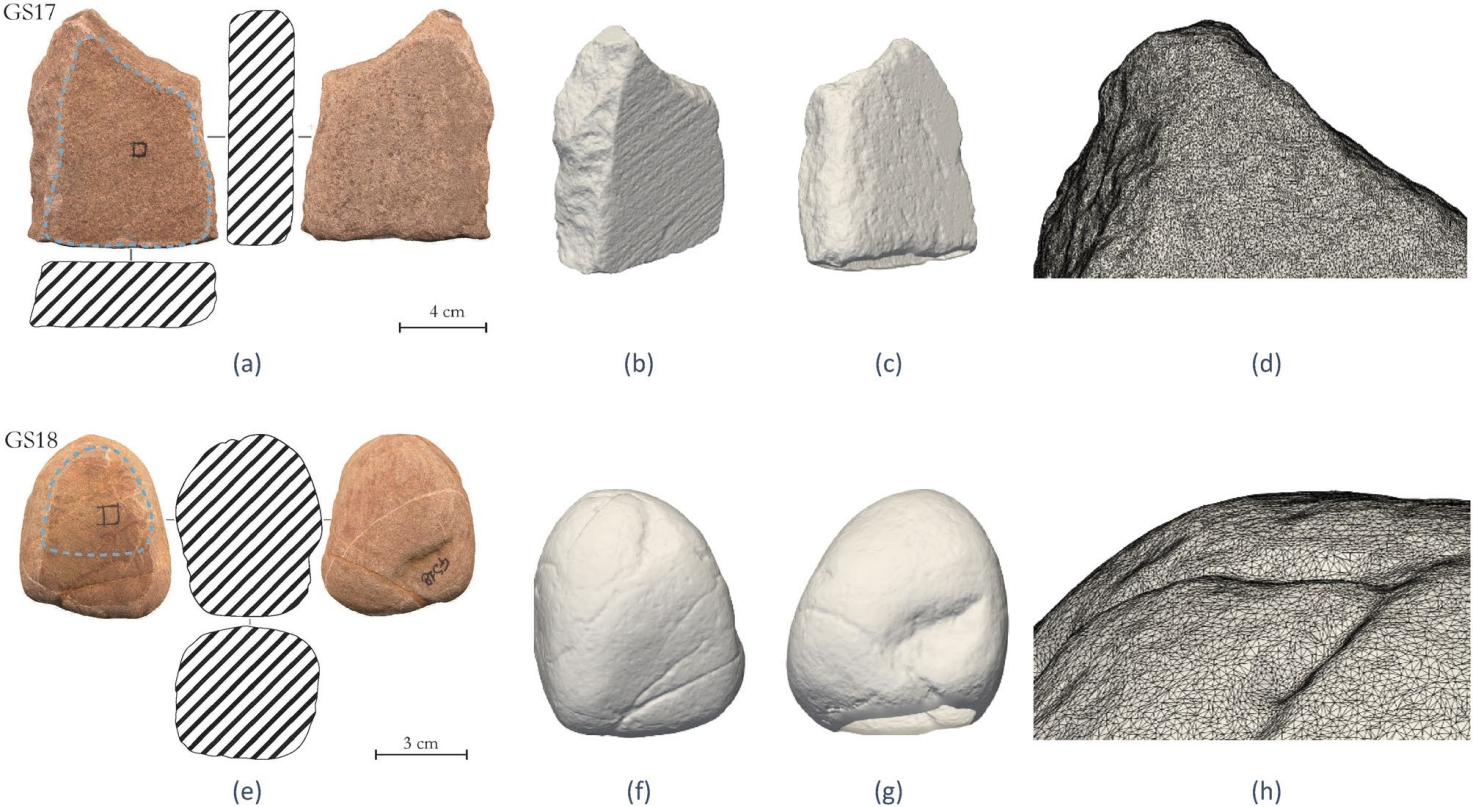
Different 3D views of the replicative ground stone tools before the experimental activity: the passive stone referred to as GS17 in (**a**–**d**) and the active tool named GS18 in (**e**–**h**). Figures (**a**) and (**e**) show the textured models, where the blue dashed lines indicate the contact areas in the experimental reproduction. The other images display the STL surface meshes. (**d**) and (**h**) show a small detail of the STL surface DT model with finite elements.

This article specifically focuses on a pair of replicated tools referred to as GS18 and GS17 which are shown in Fig. 1a and e. The stones were collected from the middle basin of the Fiora River (Manciano, Grosseto, Italy) and comprise litharenite and sandstone, respectively. GS17, used as a passive tool, has a flat trapezoidal prism shape, a length of 107.36 mm, a width of 84.09 mm, a thickness of 29.97 mm and the area designed as contact surface exhibits a flat profile and undulation at waviness level. GS18, used as an active tool, has a spherical shape, a length of 58.96 mm, a width of 42.27 mm, a thickness of 43.17 mm, and the side designed as contact surface presents a convex profile. Following rigorous cleaning procedures, the tools were employed for a duration of 2 h in *Rumex crispus* achenes processing. Processing achenes (dry fruits with shelled pericarps containing a single seed) involves grinding actions, including horizontal, bidirectional, and sometimes circular movements. The gesture can be divided into steps, starting with an initial vertical action where the two lithic surfaces come into contact and subsequently, the motion transitions to horizontal. The action is interrupted by frequent pauses to group the achenes that disperse onto the passive tool's surface upon contact with the active stone. Then the cycle of movement repeats. As a result of the medium's dispersion on the passive surface there are repeated direct contacts between the two lithic surfaces, which determine an intense surface depletion^9,35^. Data collection took place across multiple scales, involving the recurrent documentation of features prior to and after the experimental use, by acquiring 3D models and molds of the stone surface, the latest subsequently observed through various types of microscopes (for detail, see refs.^9,35^).

## Results

**Figure 2:**
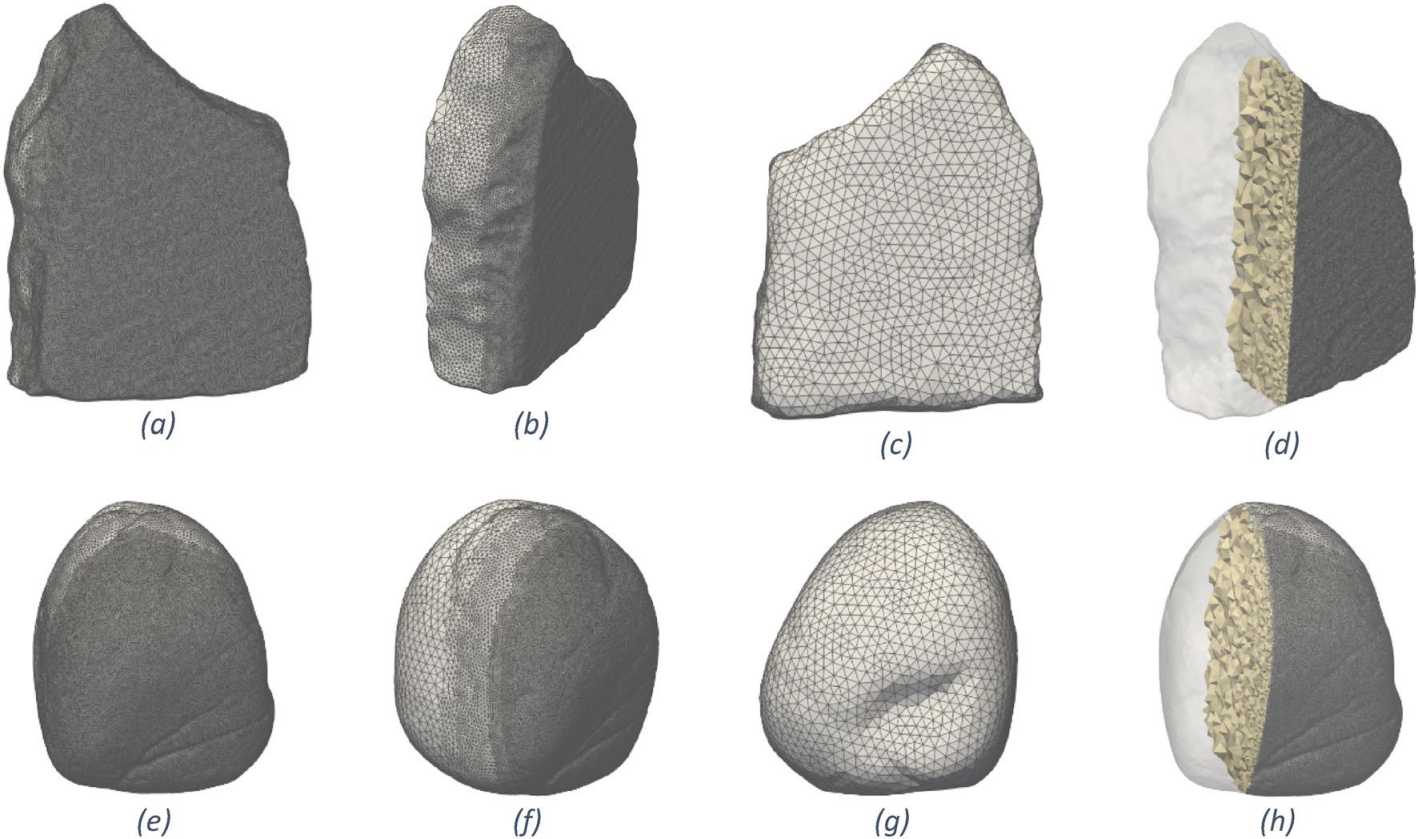
Different views and sections of the FE model of the GS17 (**a**–**f**) and GS18 (**e**–**h**) after the editing with MeshMixer and MeshLab. The mesh is finer in the contact region and degrades inside the tools (see the cross-sections (**d**) and (**h**)).

The digital model was created to replicate the damage caused by the replicative use of the stone tools and it has been devised starting from the geometrical data contained in the STL file produced with photogrammetric techniques prior to use, as detailed in^35^ and shortly summarised in the Method section. The STL surface mesh of the passive stone GS17 is shown in Fig. 1b–d, while the active stone GS18 is depicted in Fig. 1f–g. The volumetric FE mesh, obtained as described in the Method section, can be seen in Fig. 2 where it can be noticed that the volumetric mesh is finer in the area where the two stones come in contact, where the resolution is the same as in the CAD, while the mesh progressively becomes coarser in the rest of the solids where less accuracy is required.

**Figure 3:**
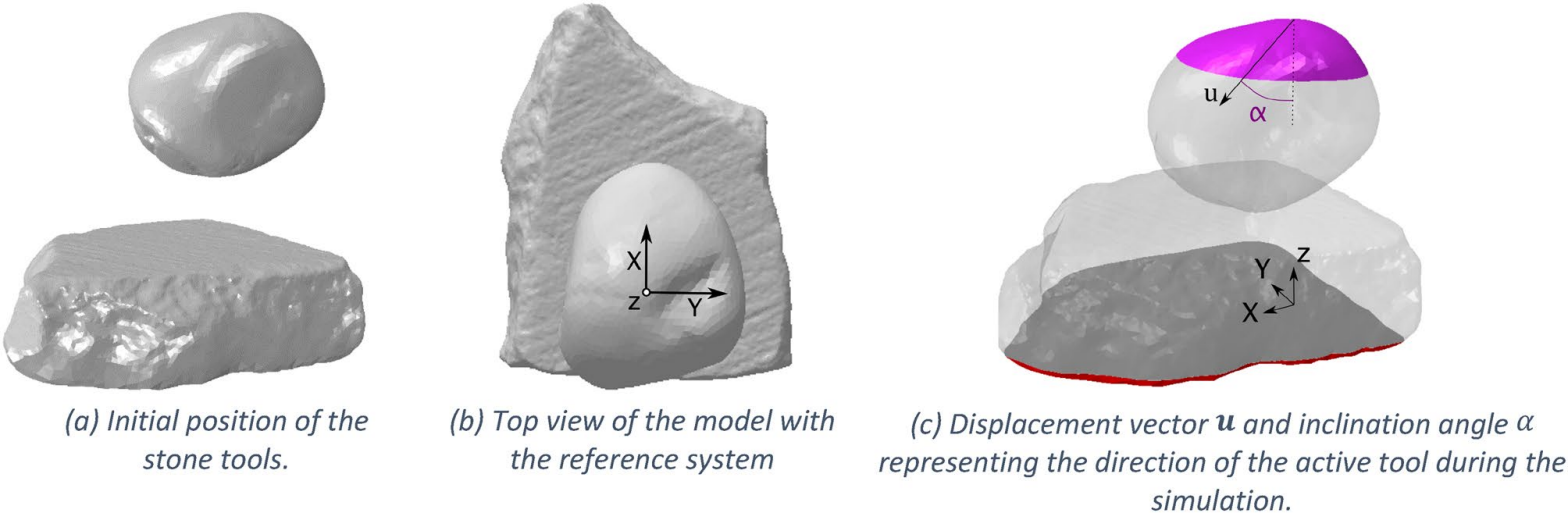
Digital twin model of the GSTs represented in different views.

Finally, the FE mesh has been imported in Abaqus where the model has been enriched with the material properties and the fracture parameters necessary to simulate the damage process of the stones using the phase-field approach for fracture, see Supplementary. The Abaqus GUI has been exploited to define the initial positions of the stones, model the contact interactions between the solids and to control the displacement to the active stone GS18 to reproduce the experimental pounding/grinding action, as shown in Fig. 3. The figure also shows the coordinate reference system and the vector ${\varvec{u}}$ that represents the direction at which the active stone is pushed against the passive stone.

The following paragraphs report results from different simulations conducted on the obtained DT to understand how damage on the tools’ surface changes with the location of the impact and the direction the active tool is pushed against the base, which are typically varying in time during the manual operation, while they are fixed in laboratory tests carried out with mechanical devices. The action has been divided in two steps: (1) the first phase consists in the motion that brings the stone tools in contact (similar to the pounding motion), where the active stone is pushed with a vertical or inclined direction towards the passive stone; (2) the second phase involves the active stone’s horizontal displacement (the proper grinding motion) on the passive stone’s surface, keeping the contact between the tools.

The first case to be analysed considers the initial phase of the gesture, simulated applying a vertical displacement $u_{z}$ (called *far-field displacement* to distinguish it from the displacements within the solids that represent the solution of the FEM problem) applied on the active stone tool; an animation of the DT model for this example is available in Supplementary Video S1 where the action is repeated in cycles only for representation purpose.

**Figure 4:**
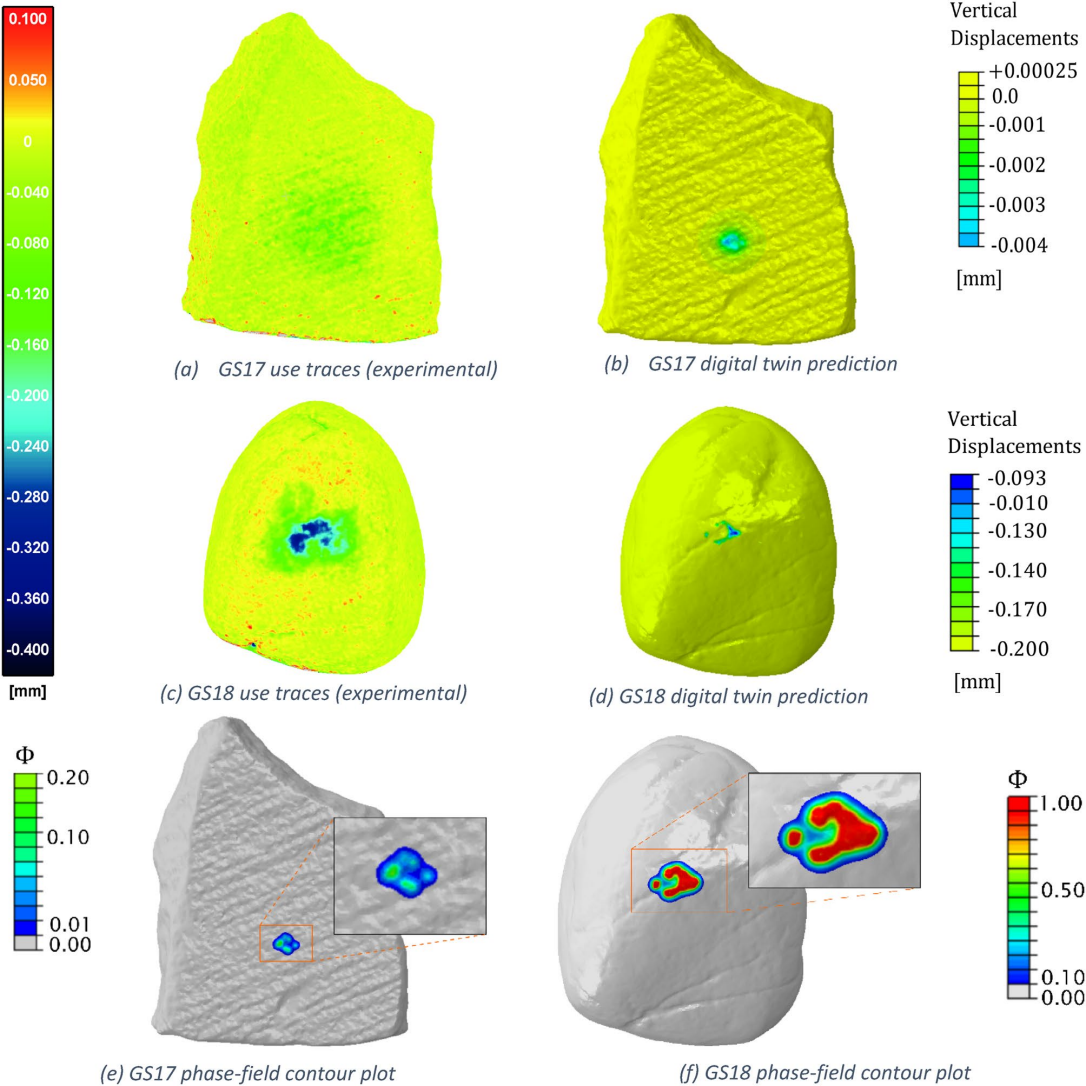
Experimental results of the active stone tools in (**a**) and (**c**) in terms of distance with respect of the photogrammetric mesh prior to the tools usage, compared to the digital twin model results in (**b**) and (**d**) represented by the vertical displacement of the FE nodes with respect to the initial position. Contour plots of the phase-field in GS17 (**e**) and GS18 in (**f**).

The deformation of the stones obtained with the DT model has been compared in Fig. 4 with the experimental damage pattern. In particular, Fig. 4a and c represent the use-traces on GS17 and GS18 using a colour map indicating the distance of the vertices of the after-use photogrammetric model to the triangles of the prior-to-use reference model. Details on the procedure exploited to map the surface damage can be found in^35^; Fig. 4b and c shows the digital twin deformation areas in terms of vertical displacement component ${u}_{3}$ of each node of the FE model.

The deformed areas match the material depletion areas analysed in^9^, even if the simulated deformed areas are smaller than the experimental counterpart because the DT reproduces only a single movement, while the experiment consisted of multiples contacts between the stones in multiple points due to the randomness of the manual operations. As already mentioned in the introduction, the FE digital twin does not only provide the deformation of the solids, but it also computes the phase-field variable $\phi$ that quantifies the damage of the stone tools. The contour plots of the phase-field variable resulting from the simulation are shown in Fig. 4e for the passive stone and in Fig. 4f for the active stone.

The DT showed that even if the stones had the same mechanical parameters, the contact causes more damage on the active tool’s surface than on the passive stone, in very good accordance with the experimental observation, where the maximum extent of depletion was up to −170 μm in the GS17 passive stone and up to −360 μm in the GS18 active stone^35^. In fact, while the phase-field parameter reaches 1 on the GS18 surface (see Fig. 4f), the damage on the GS17 surface stands at 0.1–0.2 as shown in Fig. 4e. A low damage threshold may indicate wear trace on the passive tool surface, while the phase-field value $\approx 1$ corresponds to micro-cracks, chipping or flaking on the surface. These phenomena have been noted also during the scanning process of the stones before and after the grounding operations. The evaluation of the surface roughness parameter $S_{q}$ (Root Mean Square Roughness) in^9^ showed that the operations cause the progressive decrease of roughness till a certain threshold after which $S_{q}$ slightly increases; this behaviour may indicate material removal and the creation of a new rough surface.

The DT model has been further exploited to understand if changing the point where the active stone touches the passive tool during the pounding act can affect the surface modification, since this aspect could be one of the major limitations of experimental tests using universal testing machines, which cannot easily reproduce the human variability in the gestures. GS18 has been translated from the location used in the previously described simulation, depicted in Fig. 3b, in three different positions: (1) 5 mm translation in the x direction; (2) 5 mm in the y direction; and (3) 5 mm in both x and y directions. Hence, the effect of the active tool location has been investigated by analysing the damage pattern resulting from an imposed vertical displacement $u_{z} = 0.06$ mm after the first time-step at which the stones come in contact.

**Figure 5:**
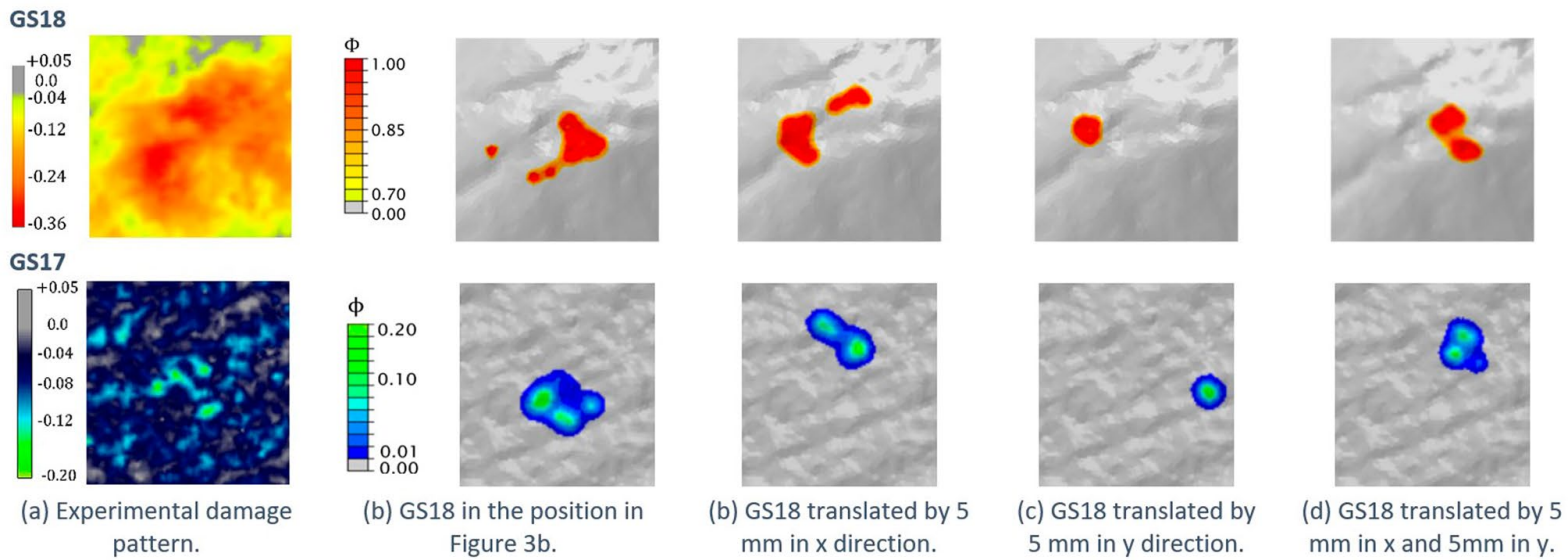
Damage patterns on GS18 surface (top) and on GS17 surface (bottom) obtained by translating the active tool position w.r.t. the position in (**b**) and imposing a far-field vertical displacement of 0.06 mm after the first point in contact.

The damage patterns for the four cases have been reported in Fig. 5 highlighting the elements with $\phi \ge 0.7$. It can be immediately noticed that the damaged portion on the GS18 surface shows a great variability in relation to the perturbation in the location of impact, which is therefore an important aspect that should be considered in testing with mechanical equipment. The comparison with the experimental results reported in Fig. 5a for both GS17 and GS18 confirmed that the simulated damage pattern is more localised than the real stone surface modification because it is caused by a single gesture and not a stochastic repetition of the same actions. This latter aspect is relevant also in case of controlled experiments, where the variability of the contact points needs to be taken into account for an accurate reproduction of the human activity and the spread of damage on the surface.

**Figure 6:**
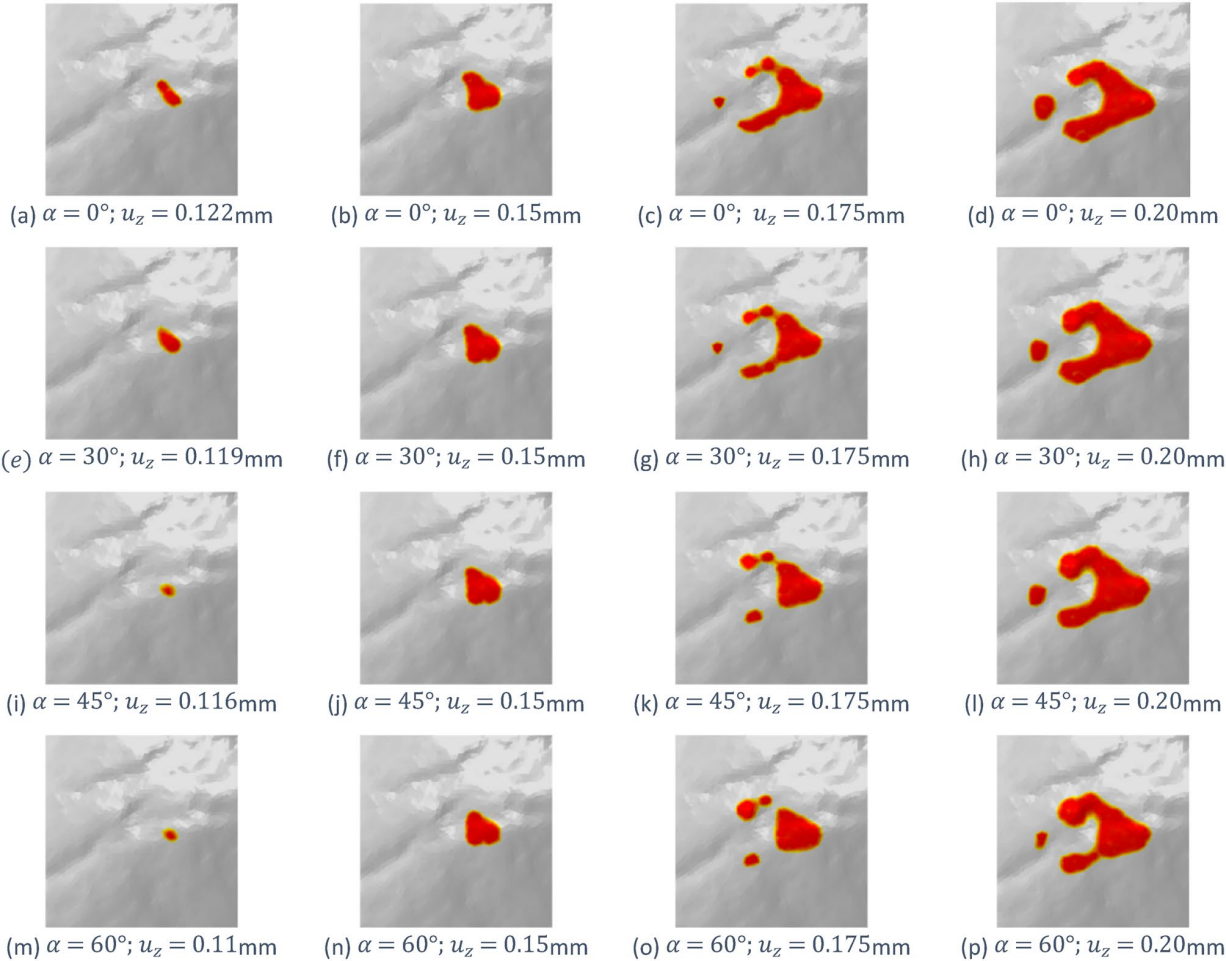
Damage pattern on the active tool GS18 surface at different time-steps, for impact angle $\mathrm{\alpha }$ = 0° (vertical direction), 30°, 45° and 60°.

A third set of simulations has been carried on starting from the initial position of the active tool and varying the angle $$\alpha$$ used to push it against the passive stone (see Fig. 3c), hence applying simultaneously far-field vertical and horizontal displacements, respectively $$u_{x}$$ and $$u_{z}$$, to the active stone to simulate an inclined stroke. The DT model has been run considering the angles α = 30°, 45° and 60° and compared to the case with α = 0° (vertical direction). As in the previous analysis, the damage pattern on GS18 shows a certain variability which can be seen in the phase-field contour plots depicted in Fig. 6. For each inclination, the contour plots are taken at the moment of damage initiation (Fig. 6a, e, h, l) and at some following time steps for the same values of far-field vertical component $$u_{z}$$$$=$$ 0,15 mm, 0.175 mm and 0.20 mm.

**Figure 7:**
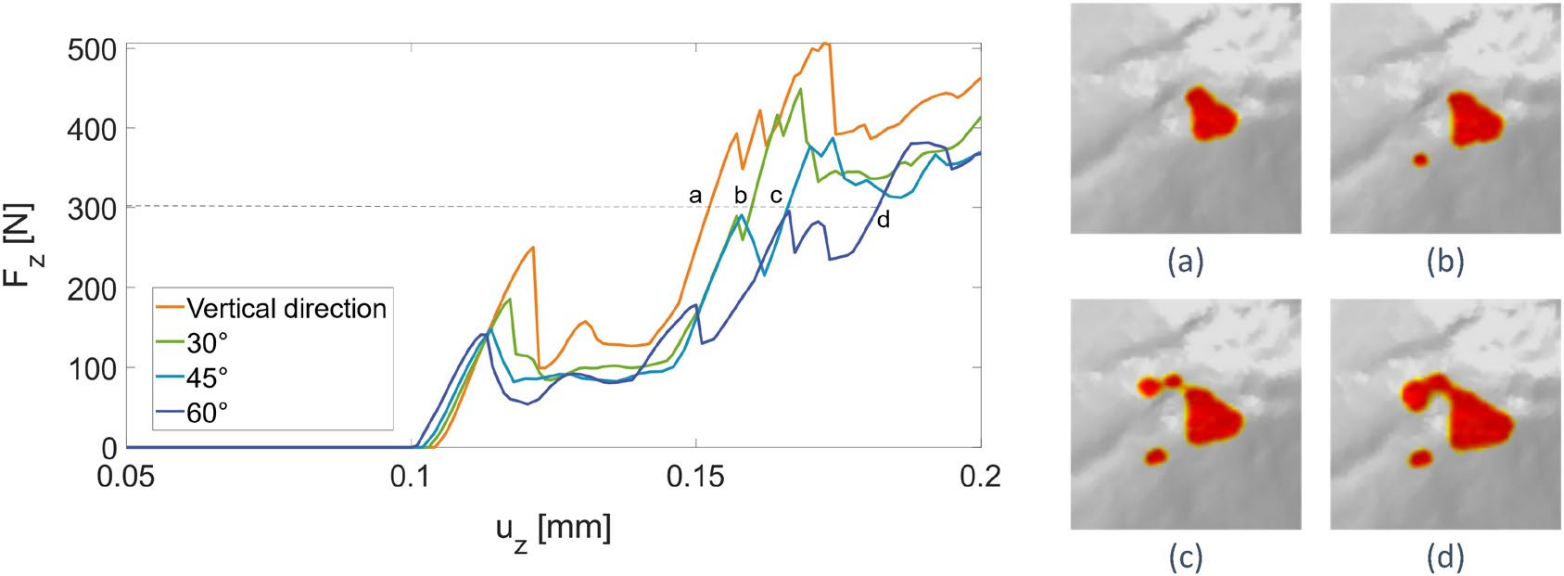
Vertical component of the total reaction force, ${\mathrm{F}}_{\mathrm{z}}$, vs. the vertical displacement ${\mathrm{u}}_{\mathrm{z}}$ applied to the active tool, for different impact directions. The contour plots on the right report the damage patterns at the points (**a**–**d**) highlighted in the plot.

The simulations have been compared also in terms of vertical reaction force, $F_{z}$ plotted in Fig. 7. Damage initiation corresponds to the first peak of the curves after the approximately linear elastic part and refers to the damage patterns reported in the first column of Fig. 7. For completeness, the horizontal reaction forces have been plotted and commented on in the Supplementary document.

Looking at the curves in Fig. 7, it can be noticed that damage starts at different values of $u_{z}$ and $F_{z}$ by perturbing the inclination angle: the force necessary to cause the first damage on the surface decreases with $\alpha$. Moreover, for the same value of $F_{z}$, the damaged portion on GS18 surface strongly varies with $\alpha$, as emphasised in the phase-field contour plots in Fig. 7 for each of the points marked on the force–displacement plot. This comparison highlights the major influence of the inclination angle in case of tests conducted under force control, which is a situation occurring during the manual usage of the active tool.

**Figure 8:**
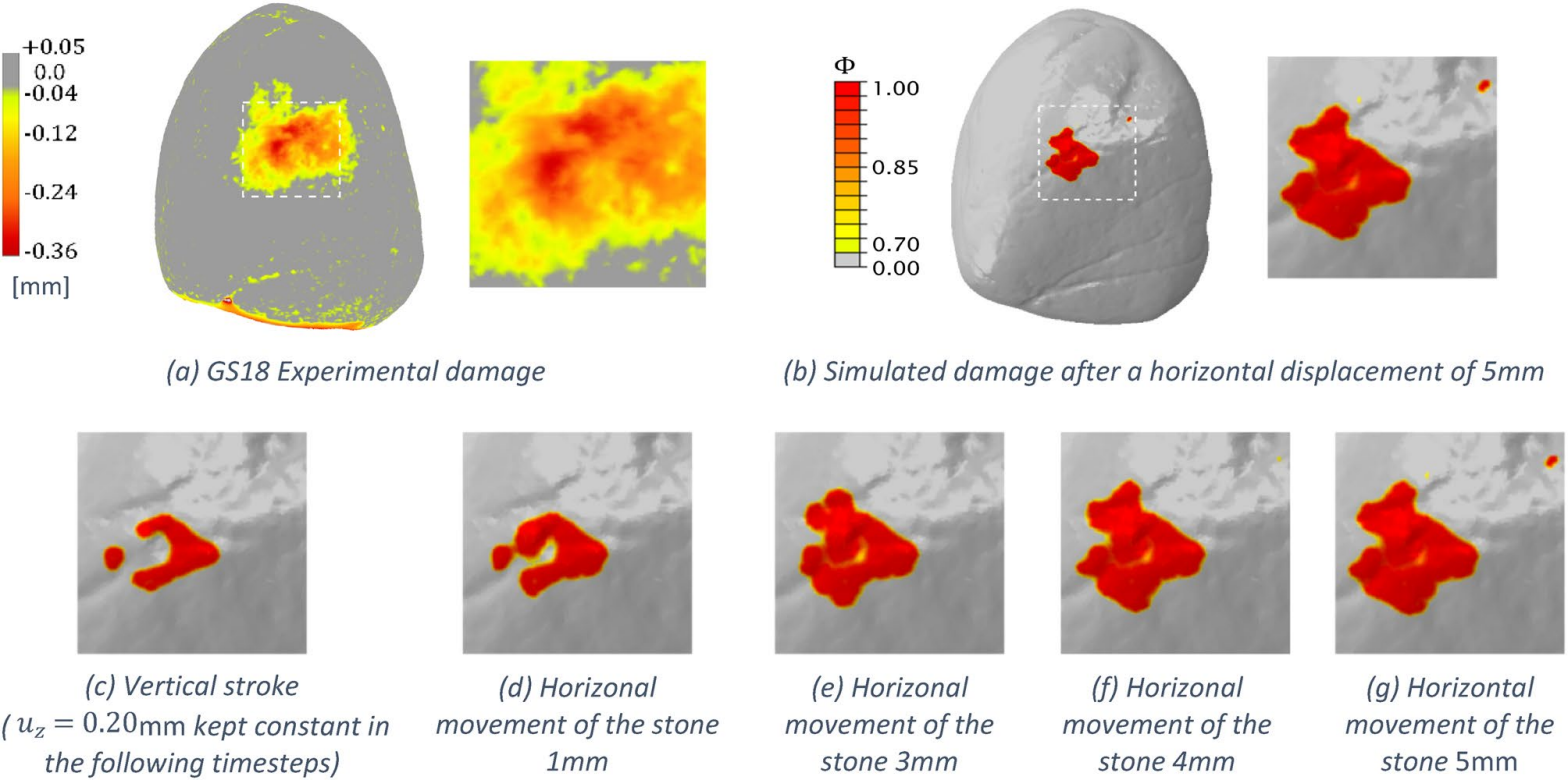
Comparison of the experimental traces on GS18 in (**a**) and damage pattern caused by the simulated grinding task (**b**). PF plots at different timesteps starting from the vertical motion in (**a**) to the maximum horizontal displacement 5 mm in (**d**–**g**).

The GSTs digital twin model can simulate the full grinding gesture, including the displacement towards the passive stone with a constant vertical motion followed by the horizontal grinding movement of the active stone. In this regard, some preliminary results are reported here to show how the surface damage evolves in this second (frictional) phase; an animation of the DT model for this example is available online as Supplementary Video S2. The simulation considers the same vertical motion previously analysed in Figs. 5, 6 and continues with a horizontal displacement in direction $x$ of 5 mm; the resulting phase-field contour plots are reported in Fig. 8 for the GS18 active stone. As shown from the figures, the initial damage area in Fig. 8c enlarges due to increasing the horizontal displacement, becoming more similar to the experimental traces left after the grinding task. This promising result opens to more in-depth analyses exploiting the developed DT.

## Discussion

This work presented the very first attempt in the literature to create a FE-based DT model for Upper Palaeolithic ground stone tools, which can be used for quantitative analyses of these archaeological items. The DT model simulates the replicas compatible with archaeological GSTs to investigate the damage patterns on the surface produced from *pounding and grinding tasks*.

A pair of experimental tools have been employed for *Rumex crispus* achenes processing, documenting the surface modifications during the experimental test through photogrammetric acquisition processes. The 3D model of the stone tools at the initial state has been used as input for the development of the DT model. The CAD surface triangulation has been edited to obtain a FE mesh optimised for achieving a good accuracy of the results with a reasonable computational time for the simulations.

The resulting DT model conjugates an accurate geometry of the stone tools and an advanced computational method to simulate damage of the tool during its use: the phase-field approach to fracture has been employed to simulate the progressive damage of the stones. The physics-based DT predicts the deformation of tools and their damage by controlling the initial position of the stones, the direction with which the active tool is pushed against the passive one, and the inclination angle of the motion. The DT model offers high control on the test settings and can be used to evaluate the effect of the variability of the human actions which cannot be easily controlled in actualistic tests or replicated within machine-controlled experimental tests.

The DT model has been exploited to simulate the manual operations, evaluating the amount of damage caused by these actions on the surfaces and comparing the damage patterns left on the physical objects during the experimental tests. The observed damage patterns significantly vary with the different configurations. This leads to an important guideline for archeologists for the design of controlled experiments under force control in the lab: the mechanical devices should consider perturbations in the location of the impact and in the inclination angle to lead to damage effects consistent with the actual manual use of the stone tools. The preliminary results on the grinding simulations showed that the DT model can capture the damage evolution due to the active stones horizontal movement which enlarges the traces left on the stones' surface with respect to the damage caused by the vertical stroke.

The presented DT model represents a first step in the direction of a more realistic DT of the GSTs, which can be enriched by considering the impact dynamics, a wear model for a more accurate simulation of the effects of cyclic movements. A third body involved in the simulated task (e.g., roots, bones, etc.) has so far neglected because the present study focuses on the first stone impact and the following grinding motion, however presence of the vegetal resources could be introduced in the DT in future investigations on wear. Moreover, by producing videos and digital reproductions of the prehistoric use of these tools, the DT model also provides an instrument to enhance the exhibition of these items in museums, increasing visitors' interest in these archaeological objects.

## Methods

This section outlines the steps necessary for the development of the digital twin model of the GSTs. First, the geometrical properties of the stone objects were captured by generating 3D models through photogrammetry, which involves capturing multiple sets of photographs that overlap with each other, taken from various positions and angles^36–38^. These images are then utilised to identify reference points, and through the triangulation process, the relative positions of these points in 3D space are determined. *Structure-from-Motion* and *Multi-View Stereo* reconstruction techniques were employed to obtain detailed 3D models of both passive and active stone tools before and after the experimental tests. The design of an ad hoc setup specifically tailored for these samples is extensively documented in^35^. It was guaranteed that each time the stones were removed from the acquisition setting for use in replicative experiments, they would be placed back in space with the same position and orientation. The acquired photographs, 114 per object, were then subjected to photogrammetric processing using Agisoft Metashape software, enabling the generation of highly refined 3D models of known accuracy. Finally, the resulting models were exported in STL format, preparing them for further analysis and visualisation (Fig. 1).

The DT model of the stones has been devised starting from the geometrical data contained in the STL file, transforming the surface triangulation in FE volume mesh discretization. The challenge here regards the fact that, even if a robust open source software like GMSH^39^ can manage the STL file, the FE meshing process is not automatic for two main reasons: (1) the presence of defects in the STL file, as holes, overlapping faces, and non-manifold edges; (2) the huge dimension of the resulting FE mesh to lead to computationally feasible simulations. Regarding the latter issue, we remark that the surface triangulation provided by the STL file resulting from the high-fidelity acquisition techniques described in the previous section is usually very fine to capture even the smallest features of the object. Since the DT model will be exploited for contact mechanics and near-surface damage simulation on specific portions of the tool boundary, the fine scale model should be retained especially on that zone.

These issues have been tackled and successfully resolved through two software for STL editing developed mainly for 3D printing applications: Autodesk MeshMixer^40^ and MeshLab^41^ to regularise the mesh, design the elements dimensions and fill the holes in the STL mesh and to deal with defects like overlapping faces and non-manifold edges.

Finally, the FE mesh has been imported in Abaqus (ver. 6.14) to simulate the damage process of the stones using the phase-field approach for fracture developed by the authors and implemented as a user element UEL subroutine, see Supplementary.

### Supplementary Information

Supplementary Information 1.

Supplementary Legends.

Supplementary Video 1.

Supplementary Video 2.

## Data Availability

The data that support the findings of this study are available from the corresponding author, M. R. M., upon reasonable request.
