# Supplementary material for: Digital twin models of replicative ground stones: insight into simulating usage of Upper Paleolithic tools
Source: Sci Rep. 2023 Oct 25;13:18298. doi: 10.1038/s41598-023-45425-4 (PMC10600171; doi:10.1038/s41598-023-45425-4)
Supplement: Supplementary file 1 — Supplementary Information 1. [file 41598_2023_45425_MOESM1_ESM.docx]

**Supplementary**

1. **Details on the finite element-based digital twin**

The FE volumetric mesh presented in the Manuscript has been imported into Abaqus (ver. 6.14), where the graphic interface can be used for pre- and post-processing tasks. The Abaqus GUI has been exploited to define the initial position of the stones (shown in Figure S1), which has been determined to imitate the manual activity performed in [1,2]. As represented in Figure S1b, the possible contact areas, which are distinguished in slave and master surface, correspond respectively to the working surface of the passive tool GS17 and of the active tool GS18 (dashed blue lines of Figure 1a and 1e of the Manuscript).

Moreover, Abaqus GUI allows to select the model boundary conditions consisting of fixing the displacement at the base of the passive stone and applying an imposed displacement $\boldsymbol{u}$ to the active tool, precisely on the GS18 upper surface (dorsal side) where the stone would be held with one hand (purple area in Figure S1c). The displacement of the active stone can be vertical or applied with a given angle with respect to the vertical axis z, reproducing an inclined stroke that pushes the stones in contact.

| 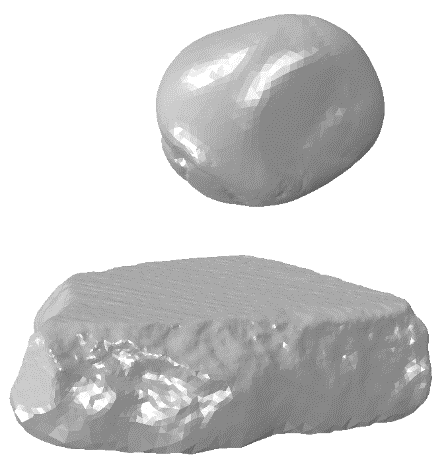 | 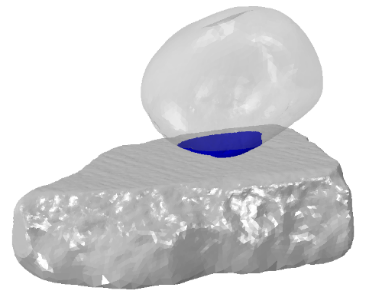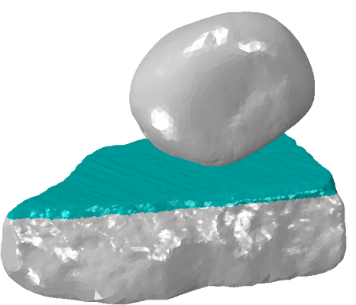 | 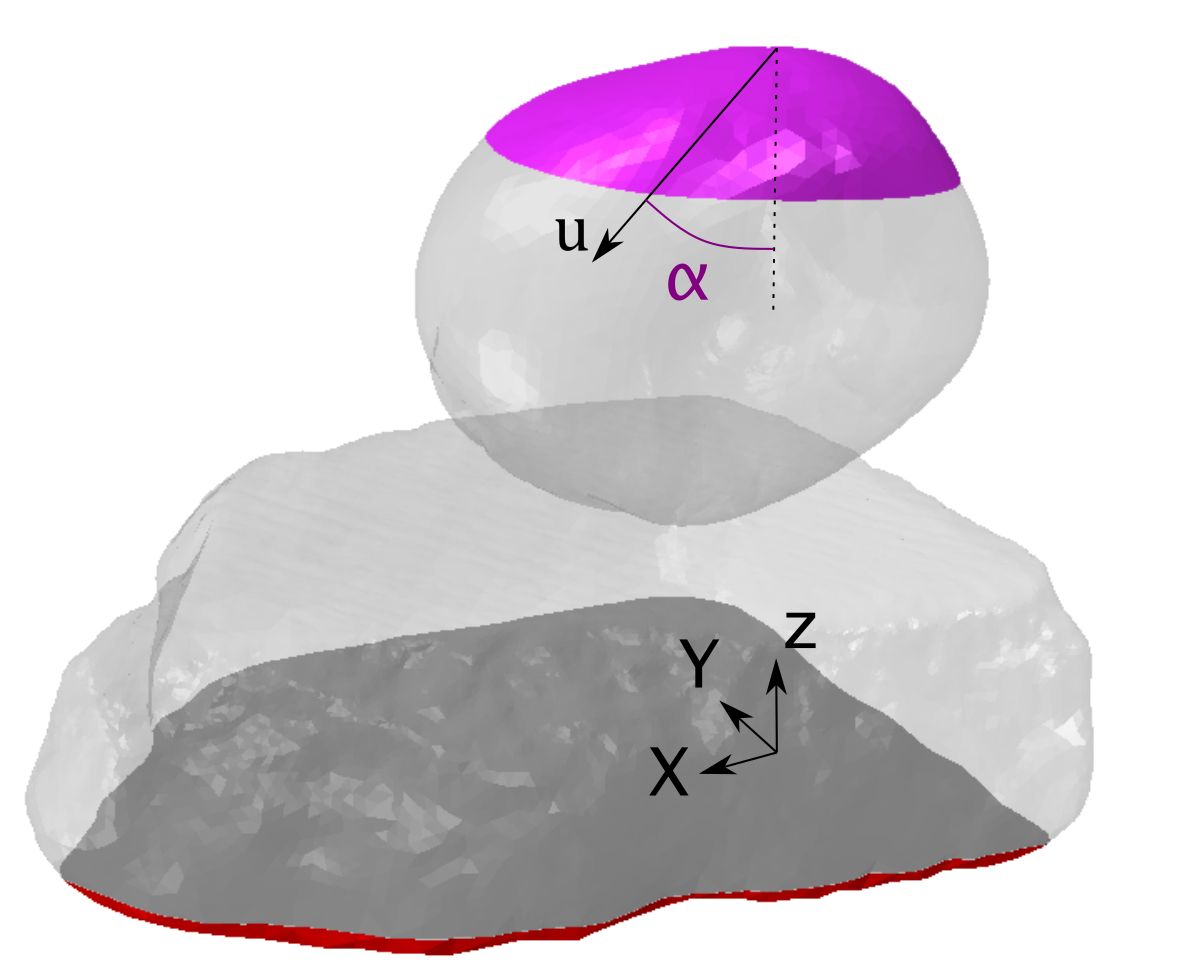 |
| --- | --- | --- |
| *(a) Digital twin model of the stone tools. The initial distance between the tools is increased here for clarity.* | *(b) Contact surfaces on GS18 (top) and on GS17 (bottom).* | *(c) Fixed area at the base of GS17 in red, and for GS18 area in purple where the displacement* $u$ *is applied with inclination* $\alpha$ *w.r.t. the vertical axis.* |

***Figure S1*** *DT model of the ground stones with contact areas and boundary conditions.*

The simulation is conducted in a quasi-static setting, increasing linearly the applied displacement in 100 timesteps. For each timestep, the Abaqus computes which nodes of the master (ventral side of the active tool) and slave (dorsal side of the passive tool) surfaces are in contact, using a standard *penalty approach* (with a value of 10^7^ N/mm for the penalty parameter). A more complex treatment of the contact problem could be explored in the future, for example, the approach formulated in [3-4], which allows to import the surface data taken from the profilometric scanning of the stone tools directly in the model, removing the need of a very fine mesh in the contact areas. The friction coefficient has been set to 0.7 as measured in [5] and used according to the Abaqus algorithm for the nonlocal frictional model described in [6].

The Abaqus UEL subroutine has been used to code a *user-defined finite element* for the damage simulation of the stones using the phase-field (PF) approach for fracture simulation.

The governing equations of the PF approach that allows to compute the displacement of each point of the stone tools, $\boldsymbol{u}=\left( u_{1},u_{2} , u_{3} \right)^{T}$, where $u_{1},u_{2} , u_{3}$ are the components in the reference system directions $x, y, z$ respectively, and the phase-field variable $\phi$ which takes values of 0 when the stone material is intact and progressively reaches 1 when it is completely broken. Starting from the pioneering works in [7-9], the PF method has been widely used to reproduce fracture nucleation and propagation of different types of materials and geometries [10-14]. This method has been applied in the presented investigation to reproduce the progressive damage, and eventually material removal, of the tools’ surfaces during the contact process.

The governing equations for a linear elastic solid $\Omega\in R^{3}$ (each stone tools) read:

$\delta\Pi_{u} =\int_{\Omega} \left( 1-\phi\right)^{2} \boldsymbol{\sigma}\left( \boldsymbol{u} \right) :\boldsymbol{\epsilon}\left( \boldsymbol{u} \right) \mathrm{dV}$ (1a)

$\delta\Pi_{\phi} =\int_{\Omega} 2\left( 1-\phi\right) \psi\left( \boldsymbol{\epsilon} \right) \delta\phi\mathrm{dV} -\int_{\Omega} G_{c} \left( \frac{\phi}{l_{0}} \delta\phi+l_{0}\nabla\phi\cdot\nabla\delta\phi\right)\mathrm{dV}$ (1b)

In the given equations, the strain and stress tensors have been noted respectively as $\boldsymbol{\epsilon}\left( \boldsymbol{u} \right)$ and $\boldsymbol{\sigma}\left( \boldsymbol{u} \right)$, while the elastic strain energy is indicated with $\psi\left( \boldsymbol{\epsilon} \right).$ $G_{c}$ represents the fracture energy, the critical value of energy release rate for which the crack propagates, and it has been considered equal to 0.0070 N/mm taking into account the fracture toughness value given in [15], and considering that the crushing energy can be estimated as two orders of magnitude smaller than the critical fracture energy for tensile fracture [12].

Another needed parameter is the length scale, which has been computed using the standard formula $l_{0}=\frac{27}{256}\frac{G_{c}E}{\sigma_{c}^{2}}=0.57$mm, considering the Young Modulus of the stones $E=19.5$ GPa and the compressive strength of the stone, $\sigma_{c}$ equal to 50.40 MPa given in [15], that reports the mechanical properties of a Floresta sandstone, because the mechanical characterization of the stones in [1, 2] are not yet available and will be introduced in the model in future extension of the DT. The Poisson ratio, needed for computing $\boldsymbol{\sigma}\left( \boldsymbol{u} \right)$, has been set equal to 0.24 as in [15].

In the specialized literature [7-14], the individual phase-field formulations differ for the degradation function used to lower the damaged material stiffness and for the method used to enforce the irreversibility condition (the level of damage can only increase). In the present work, the phase-field approach has been implemented considering the standard AT2 approach proposed in [9]. The fracture simulation has been conducted assuming that all the deformation components are equally affected by the damaged development, including the damage in compression, which is the predominant phenomenon in this case.

1. **Results for the inclined stroke simulation**

The following paragraphs expand the results presented in the Results section of the Manuscript regarding the case of the stroke inclined with respect to the vertical axis. Three cases have been considered: $\alpha=30^{\circ}, 45^{\circ}, 60^{\circ}$ and the related damage patterns have been reported in Figures 6 and 7 of the Manuscript.

| 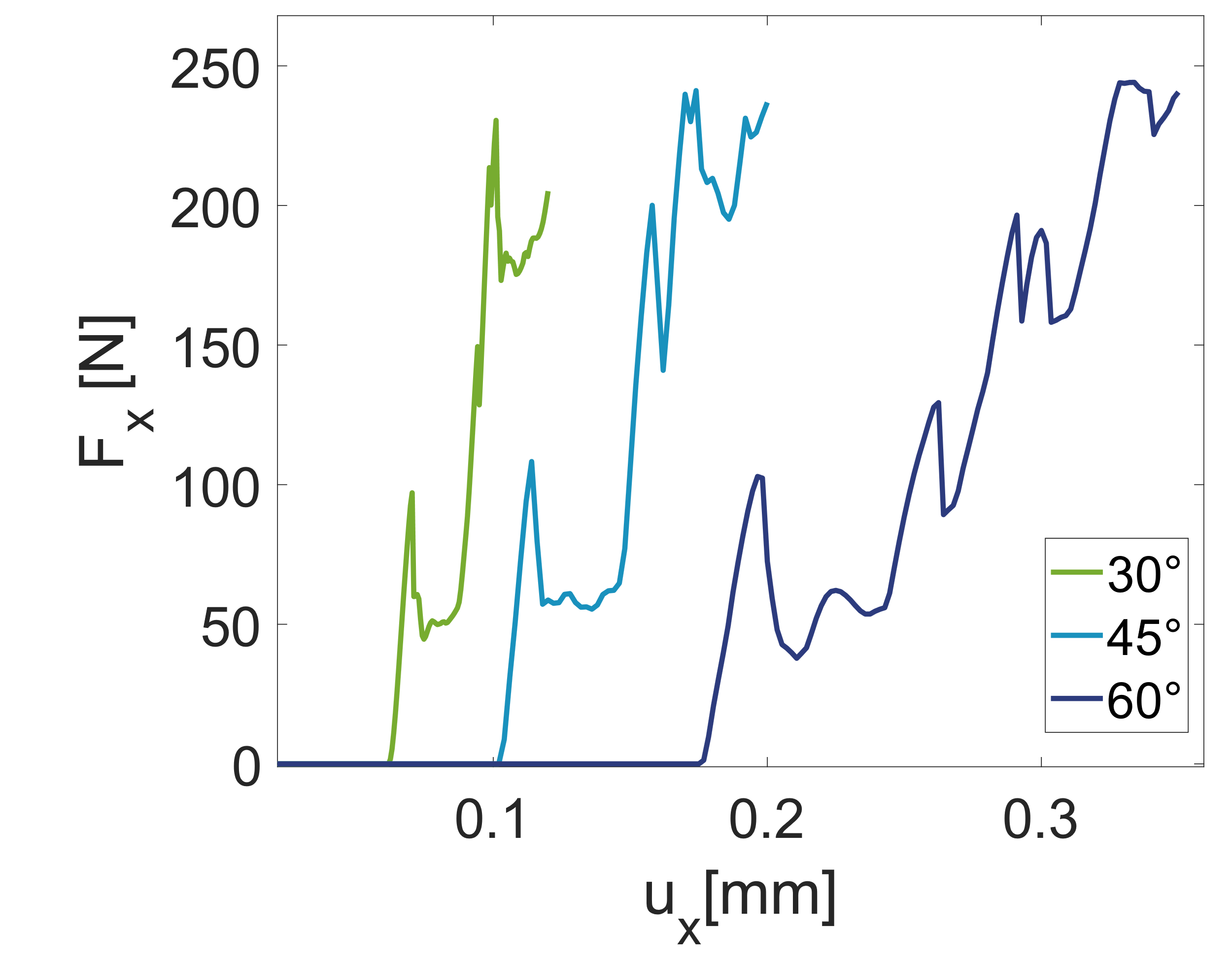 | 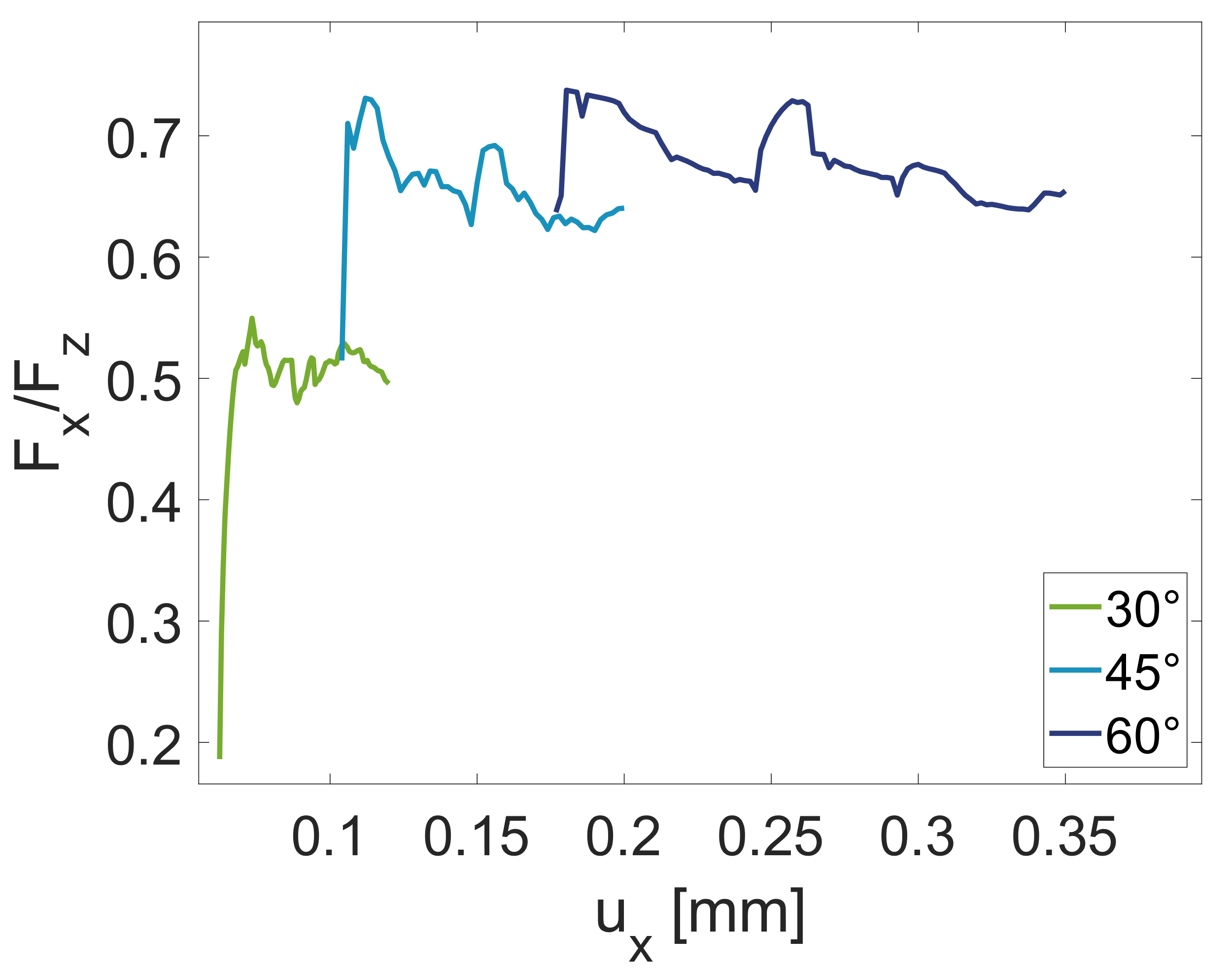 |
| --- | --- |
| (a) | (b) |

***Figure S2*** *Horizontal component of the total reaction force* $F_{x}$ *(a), and ratio of the horizontal component and the vertical one* $F_{x}/F_{z}$ *in (b) vs. the horizontal displacement* $u_{x}$ *applied to the active tool, for different impact directions.*

The horizontal component of the total reaction force $F_{x}$ has been plotted in Figure S2a vs. the imposed horizontal displacement $u_{x}.$In this case, damage initiation occurs around the same value of the force $F_{x}$, on the contrary of what is seen for $F_{z}$ which is more affected by the inclination angle. The ratio of the horizontal component to the vertical one is depicted in Figure S2(b) and shows the frictional behavior of the active tool on the passive stone. For the smallest inclination angle, the simulated emergent macro-scale friction coefficient stands at about 0.5, different from the micro-scale coefficient given as input in the simulation, $\mu=0.7,$ while this value is retrieved from the inclinations angle $\alpha=45^{\circ}$ and 60°. Hence, we can state that the frictional behavior is importantly affected by the inclination angle as well.

**References**

1. Sorrentino, G., et al., Tracing Old Gestures: A Multiscale Analysis of Ground Stone Tools Developed on Sequential Lab-Controlled Replicative Experiments. Heritage, 6, 4737–4767, (2023).
2. Sorrentino, G., et al., Close-range photogrammetry reveals morphometric changes on replicative ground stones, PLoS One, 18 (2023).
3. Marulli, M. R., Bonari, J., Reinoso, J., Paggi, M., A coupled approach to predict cone-cracks in spherical indentation tests with smooth or rough indenters, J. Mech. Phys. Solids, 178, 105345 (2023).
4. Bonari, J., Paggi, M., Reinoso, J., A framework for the analysis of fully coupled normal and tangential contact problems with complex interfaces, *Finite Elem. Anal. Des.*, **196**, (2021).
5. E. H. Rutter and C. T. Glover, The deformation of porous sandstones; are Byerlee friction and the critical state line equivalent?, J. Struct. Geol., 44, 129–140 (2012).
6. Oden, J. T., and E. B. Pires, Nonlocal and Nonlinear Friction Laws and Variational Principles for Contact Problems in Elasticity, Journal of Applied Mechanics, 50, 67–73 (1983).
7. G. A. Francfort and J.-J. Marigo, Revisiting brittle fracture as an energy minimization problem, *J. Mech. Phys. Solids*, **46**, 1319–1342 (1998).
8. B. Bourdin, G. A. Francfort, and J.-J. Marigo, Numerical experiments in revisited brittle fracture, *J. Mech. Phys. Solids*, **48**, 797–826 (2000).
9. C. Miehe, F. Welschinger, and M. Hofacker, Thermodynamically consistent phase-field models of fracture: Variational principles and multi-field FE implementations, Int. J. Numer. Methods Eng., 83, 1273–1311 (2010).
10. J. Y. Wu, V. P. Nguyen, C. T. Nguyen, D. Sutula, S. Sinaie, and S. P. A. Bordas, Phase-field modeling of fracture, Advances in Applied Mechanics, 53, 1–183 (2020).
11. M. R. Marulli, A. Valverde-González, A. Quintanas-Corominas, M. Paggi, and J. Reinoso, A combined phase-field and cohesive zone model approach for crack propagation in layered structures made of nonlinear rubber-like materials, Comput. Methods Appl. Mech. Eng., 395, 115007 (2022).
12. P. Lenarda, J. Reinoso, and M. Paggi, Multi-phase field approach to tensile fracture and compressive crushing in grained heterogeneous materials, Theor. Appl. Fract. Mech., 122, 103632, (2022).
13. M. Paggi, M. Corrado, and J. Reinoso, Fracture of solar-grade anisotropic polycrystalline Silicon: A combined phase field–cohesive zone model approach, *Comput. Methods Appl. Mech. Eng.*, **330**, 123–148 (2018).
14. P. Lenarda, J. Reinoso, and M. Paggi, Multi-phase field approach to tensile fracture and compressive crushing in grained heterogeneous materials, *Theor. Appl. Fract. Mech.*, **122**, 103632, (2022).
15. J. Justo, J. Castro, M. Miranda, D. Gatica, and S. Cicero, The theory of critical distances applied to fracture of rocks with circular cavities, *Theor. Appl. Fract. Mech.*, **121** (2022).
