# Supplementary material for: Digital twin models of replicative ground stones: insight into simulating usage of Upper Paleolithic tools
Source: Sci Rep. 2023 Oct 25;13:18298. doi: 10.1038/s41598-023-45425-4 (PMC10600171; doi:10.1038/s41598-023-45425-4)
Supplement: Supplementary file 2 — Supplementary Legends. [file 41598_2023_45425_MOESM2_ESM.docx]

**Supplementary Video S1 Label**

GSTs digital twin model animation simulating the gesture with which the active stone is pushed against the passive stone with a vertical displacement at the beginning of the grinding action. The action is repeated in cycles only for representation purpose.

**Supplementary Video S2 Label**

GTSs digital twin model animation simulating the grinding action including the active stone vertical displacement that brings the stones in contact, and the subsequently horizontal motion. The action is repeated in cycles only for representation purpose.
